# Usefulness of an Image Fusion Model Using Three-Dimensional CT and MRI with Indocyanine Green Fluorescence Endoscopy as a Multimodal Assistant System in Endoscopic Transsphenoidal Surgery

**DOI:** 10.1155/2015/694273

**Published:** 2015-08-03

**Authors:** Akihiro Inoue, Takanori Ohnishi, Shohei Kohno, Naoya Nishida, Yawara Nakamura, Yoshihiro Ohtsuka, Shirabe Matsumoto, Shiro Ohue

**Affiliations:** ^1^Department of Neurosurgery, Ehime University School of Medicine, 454 Shitsukawa, Toon, Ehime 791-0295, Japan; ^2^Department of Otolaryngology, Ehime University School of Medicine, 454 Shitsukawa, Toon, Ehime 791-0295, Japan

## Abstract

*Purpose*. We investigate the usefulness of multimodal assistant systems using a fusion model of preoperative three-dimensional (3D) computed tomography (CT) and magnetic resonance imaging (MRI) along with endoscopy with indocyanine green (ICG) fluorescence in establishing endoscopic endonasal transsphenoidal surgery (ETSS) as a more effective treatment procedure. *Methods*. Thirty-five consecutive patients undergoing ETSS in our hospital between April 2014 and March 2015 were enrolled in the study. In all patients, fusion models of 3D-CT and MRI were created by reconstructing preoperative images. In addition, in 10 patients, 12.5 mg of ICG was intravenously administered, allowing visualization of surrounding structures. We evaluated the accuracy and utility of these combined modalities in ETSS. *Results*. The fusion model of 3D-CT and MRI clearly demonstrated the complicated structures in the sphenoidal sinus and the position of the internal carotid arteries (ICAs), even with extensive tumor infiltration. ICG endoscopy enabled us to visualize the surrounding structures by the phasic appearance of fluorescent signals emitted at specific consecutive elapsed times. *Conclusions*. Preoperative 3D-CT and MRI fusion models with intraoperative ICG endoscopy allowed distinct visualization of vital structures in cases where tumors had extensively infiltrated the sphenoidal sinus. Additionally, the ICG endoscope was a useful real-time monitoring tool for ETSS.

## 1. Introduction

Endoscopic endonasal transsphenoidal surgery (ETSS) has become a more common procedure in the treatment of midline skull-based lesions such as pituitary tumors [1–6]. ETSS offers several technical advantages, including wider, multidirectional views of the operative field [7, 8]. In spite of these advantages, there is significant risk of injury to the surrounding vital structures, particularly when the tumors have extensively invaded into the sphenoidal sinus and when the bony components of the skull base have been destroyed. In such cases, it is critical to identify and monitor the vital structures by both preoperative simulation and intraoperative real-time detection.

To date, we have reconstructed 3D-CT models from 2D-CT images to allow more accurate visualization of the anatomical orientation of the nasal cavity and paranasal sinuses. With these models, we can precisely locate vital structures, such as the internal carotid arteries (ICAs) and optic nerves, as well as the anatomical relationships between these structures and the sellar floor. However, in cases where the tumor occupies the paranasal sinus, where normal bony structures have been destroyed, or where the ICAs are buried in the tumor, only 3D-CT images are insufficient for identifying the exact location of the ICAs. In other cases, where the tumor has extensively infiltrated the cavernous sinus and largely extends into the suprasellar region, it becomes very difficult to identify the ICAs and the normal pituitary gland. To overcome these problems, we created a fusion model of 3D-CT and MRI, which can demonstrate the positional relationship between the tumor and the sellar structures, including the ICAs.

Recently, fluorescence angiography using an indocyanine green (ICG) microscope has been utilized to confirm real-time blood flow [9–11]. Furthermore, angiography with an ICG endoscope has been shown to be useful in verifying the patency of vessels hidden from microscopic view during ETSS [12–16]. In the present study, we investigate the usefulness of a fusion model of 3D-CT and MRI along with an ICG endoscope as a multimodal assistant system in ETSS, particularly in cases requiring identification of the ICAs for safe and maximum resection of the tumor.

## 2. Materials and Methods

All procedures performed in studies involving human participants were in accordance with the ethical standards of the institutional and/or national research committee and with the 1964 Helsinki Declaration and its later amendments or comparable ethical standards. The present study was approved by the local ethics committee for clinical research.

Thirty-five patients with sellar or intrasphenoidal sinus tumors were treated with ETSS at Ehime University Hospital from April 2014 to March 2015. They included patients with pituitary macroadenoma (tumor size ≥10 mm), craniopharyngioma, Rathke's cleft cyst, chordoma, and giant cell tumor. Neurological and endocrinological examinations were assessed preoperatively as well as immediately after surgery and at 3 months later. All patients had visual examinations including visual acuity and visual field assessed by Goldmann perimeter. Standard hormonal evaluation was performed in all patients. The diagnosis of acromegaly was based on clinical symptoms and the endocrinological requirements, consisting of an elevated level of insulin-like growth factor 1 (IGF-1) compared to the age- and gender-matched value and insufficient suppression of growth hormone (GH) after loading 75 g oral glucose. The patient with prolactin (PRL) producing adenoma was involved in the study because she could not take a dopamine agonist due to her myocardial disturbance. The radiologist created a fusion model from the reconstructed preoperative 3D-CT and MRI of all patients. In addition, from January 2015, ICG endoscope was introduced in our department. By the subsequent operation, during ETSS, ICG was administered intravenously to the remaining 10 patients, and vital structures, including the ICAs, were visualized via the ICG endoscope.

Details of these methods were described in the paragraphs that follow. Informed consent was obtained from all individual participants enrolled in the study, including the surgical procedure and potential risks of ETSS.

### 2.1. Fusion Model of Reconstructed 3D-CT and MRI

MRI and CT scans were performed before surgery on each patient. Contrast-enhanced CT was performed immediately after injection of 70 mL of intravenous iodinated contrast medium (Omnipaque Injection, 350 mg I/mL; Daiichi Sankyo Company, Tokyo, Japan) using a 64-multidetector row CT (Brilliance 64; Philips Healthcare, Andover, MA, USA and Best, Netherlands). The collimation was 64 × 0.625 mm and the gantry rotation time was 0.4 s. Scan parameters were as follows: voltage, 120 kV; tube current, 300 mAs (228 mA); field of view (FOV), 250 mm; matrix, 512 × 512; and Brain Smooth (UV) filter. MRI studies were performed using a 3.0-T whole-body MR scanner (Gyroscan Achieva; Philips Medical Systems, Best, Netherlands) and an eight-channel phased-array head coil. For each patient, a high-resolution anatomic data set was scanned using 3D spoiled gradient-recalled echo sequences (repetition time, 15 ms; echo time, 2.3 ms; flip angle, 10; matrix, 256 × 320; FOV, 230 mm; thickness, 0.9 mm) with gadopentetate dimeglumine.

Image processing of the acquired CT and MRI was performed using a 3D Advantage Workstation Volume Share 4 (GE Healthcare, Waukesha, WI, USA). Step 1: render volume and define opacity −250~+600 as skin parameter. Step 2: set the FOV to 18 cm and adjust the pituitary gland to the center of the monitor with other images (either axial, coronal, or sagittal). Step 3: set position and select the volume rendering Front Cut function (squarely whittle at image) and then cut from the tip of the nose to the end of virus ring. Step 4: confirm position and scope and then save. Step 5: overlay the CT volume-rendered (VR) image (CTA image) obtained from the above process onto the MRA image and the VR image obtained from contrast-enhanced MRI. Step 6: for overlaying the image, perform (1) autofusion (overlay an image with automatic adjustment of FOV and position information) or (2) manual fusion (overlay an image while checking the image and adjusting the triaxial *X*-*Y*-*Z*). Step 7: confirm fusion image and save.

Acquired fusion image model data from MRI and 3D-CT were stored in Digital Imaging and Communications in Medicine (DICOM) format. A slide presentation file was prepared from digital snapshots taken at different phases of the simulated operation, and this file was used as a visual reference during the actual surgery. Based on images from the fusion model, we evaluated the structure of the sphenoidal sinus and location of the vital structures, including the bony prominences of the ICAs and optic canals buried under the invasive tumor, and made comparisons with the actual endoscopic views observed intraoperatively.

### 2.2. Neuronavigation System

We used the surgical navigation system (StealthStation S7, Medtronic Inc., Louisville, CO). The system has both optical type and magnetic field type and it is possible to choose a system according to an operative situation. For ETSS in the present study, magnetic tracking mode was usually used. The magnetic sensor was fixed to a mouthpiece that was made to order for each patient. Preoperative CT and MR images were taken for the navigation system and these data were transferred to the workstation of the navigation system.

### 2.3. ICG Fluorescence

The ICG compound (25 mg), purchased from Daiichi-Sankyo, was dissolved in 10 mL of sterile water. In 10 patients, 5 mL of the solution (12.5 mg of ICG) was injected into a peripheral vein as a bolus during surgery, which was followed by flushing with 10 mL of saline. The maximum absorption and emission wavelengths of ICG in water are 780 nm and 805 nm, respectively; in plasma, they are 800 nm and 825 nm, respectively [17].

### 2.4. Endoscope and ICG Endoscope

Two different endoscopes were used for the present surgery. One was a rigid 0°, 30°, and 70° endoscope (4 mm in diameter, 11 cm in length; Olympus, Japan) that was used for ETSS with or without EndoArm assistance. (Olympus). The other was an ICG endoscope with the following features: a straightforward 0° telescope (5.8 mm in diameter, 19 cm in length), a TRICAM PDD 3-Chip Camera Head, a TRICAM SLII 3-Chip Camera Control Unit, and a Cold Light Fountain D-LIGHT P (all from Karl Storz) [12]. The light source could be converted from white light to near-infrared light with a foot switch. A TRICAM PDD system was calibrated by white balancing under white light with no filter. We placed the ICG endoscope in a suitable position under white light and then switched to near-infrared light, whereupon the fluorescent signals from the ICG flow in the ICAs became visible.

### 2.5. Statistical Methods

Statistical analysis was performed using Fisher's exact test for categorical variables and analysis of variance for continuous variables. Two-tailed tests were performed for each scenario, and the significance level was set at *P* < 0.05. All analyses were performed using Office Excel 2013 software (Microsoft, Redmond, WA, USA).

## 3. Results

### 3.1. Preoperative Patient Characteristics

35 patients in the present study included those with pituitary adenomas (*n* = 27), craniopharyngioma (*n* = 3), Rathke's cleft cyst (*n* = 3), chordoma (*n* = 1), and giant cell tumor (*n* = 1) (Table 1). The hormonal types of the patients with pituitary adenoma were nonfunctioning adenoma (*n* = 22), GH producing adenoma (*n* = 4), and PRL producing adenoma (*n* = 1). Mean age at the time of surgery was 55.5 years (range, 16–84 years). Subjects comprised 17 women (48.6%) and 18 men (51.4%). No significant difference in age was evident between male and female patients (*P* > 0.05). Preoperatively, visual impairment was seen in 14 patients of pituitary adenomas, one of craniopharyngioma, and one of Rathke's cleft cyst. Two patients of pituitary adenomas, two of Rathke's cleft cyst, and all three of craniopharyngiomas showed deficiency of anterior pituitary hormones (Table 2). All patients were operated on by ETSS alone under general anesthesia.

### 3.2. Postoperative Outcome

Surgery was performed in all 35 patients without serious complications such as ICA injury. One patient showed cerebrospinal rhinorrhea that required repair surgery, decreased visual function was seen in one, and one needed hormone replacement therapy. Histological examination showed GH expression in four GH producing adenoma, PRL expression in one PRL producing adenoma, and no expression of all pituitary hormones in 22 nonfunctioning adenoma. MIB-1 labeling indices of these pituitary adenomas ranged from 0.6 to 1.8%. Histopathology of craniopharyngioma and Rathke's cleft cyst revealed a cytokeratin-positive adamantinomatous type and a feature of columnar epithelium, respectively. Postoperative MRI showed sufficient resection of the tumors with no or little evidence of residual tumor tissue in 33 patients except for the patients with chordoma and giant cell tumor. Three patients of four GH producing adenomas showed normalization of GH secretion, and in the patient with PRL producing adenoma the level of PRL decreased to 33.5 ng/mL.

Visual disturbance was immediately improved after surgery in eleven patients, but only one patient with pituitary adenoma showed deteriorated visual function after surgery (Table 2). One patient with craniopharyngioma was in need of all pituitary hormones including antidiuretic hormone after surgery.

### 3.3. Fusion Models of 3D-CT and MRI

**Figure 1:**
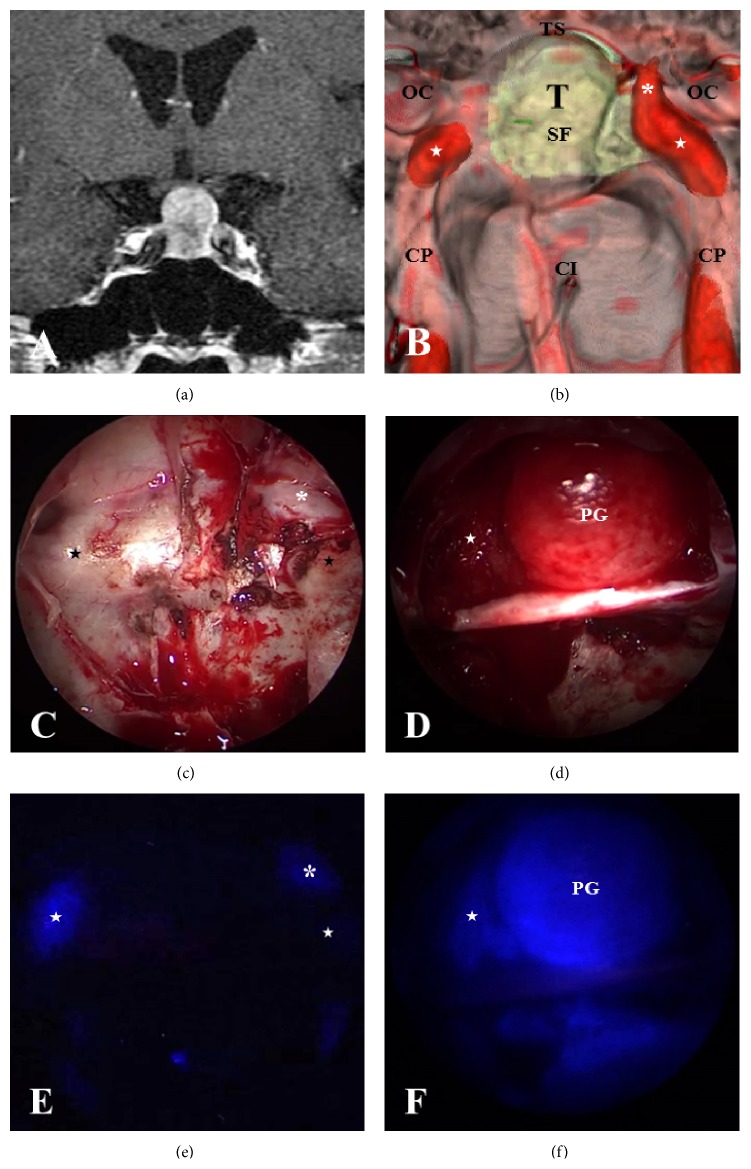
An illustrative case showing images of a fusion model of 3D-CT and MRI and intraoperative ICG endoscopy along with the corresponding endoscopic views. (a) Gadolinium- (Gd-) enhanced magnetic resonance imaging (MRI) showing a pituitary adenoma with suprasellar extension (coronal section). (b) Preoperative simulated view of the endoscopic endonasal transsphenoidal approach produced by the fusion model of 3D-CT and MRI that show bony prominences of the internal carotid arteries (ICAs), optic canals, and pituitary tumor around the sellar floor. (c) Intraoperative endoscopic view showing bony structures around the sellar floor (unopened). (d) Intraoperative endoscopic view showing the intrasellar structures, mainly the descending diaphragma sellae (DS) with the normal pituitary gland after total resection of the tumor. (e) Indocyanine green (ICG) endoscope showing vital structures such as the ICAs (a view corresponding to (c)) and (f) ICG endoscope showing ICAs in the right cavernous sinus and a normal pituitary gland adhered to the DS (SF: sellar floor; TS: tuberculum sella; CI: clival indentation; CP: ICA prominence; stars: C4 portion of ICA; asterisks: C3 portion in the cavernous sinus; OC: optic canal; and T: tumor). (e) Before opening the sella; (f) after tumor resection.

We successfully created a fusion model of 3D-CT and MRI in all patients. In the model, critical structures such as the ICAs, optic canals, the pituitary gland, and the tumor were completely reconstructed, and their spatial relationships were better visualized by successively deleting adjacent bony structure images. The intraoperative anatomy was consistent with the preoperative simulation that had been made based on the fusion models (Figures 1(a) and 1(b)). However, two major issues can complicate visualization of the anatomical structures of the sphenoidal sinus: the presence of multiple Onodi (sphenoethmoidal) cells and an invasive tumor mass occupying the sphenoidal sinus. The former was observed in 6 patients (17.1%), and the latter was observed in 7 patients (20.0%). The models revealed the entire course (C3–C5) of the bilateral ICAs and the intracranial arteries by successively eliminating images of the sphenoidal bony structures from anterior to posterior, even if the ICAs were involved in the tumor. The optic prominences were also identified in all patients. Onodi cells were clearly recognized in the models. Furthermore, we clearly recognized the positional relationship between the ICAs and invasive tumor. The fusion model of 3D-CT and MRI was possible to be viewed in the operating room during surgery as multislice presentations on a computer monitor. The images of CT/MRI fusion model were not created to be utilized for neuronavigation system. Compared to 2D images and 3D-CT models alone, the fusion model had the advantage of presenting images that much more clearly delineated the anatomical relationships between the sella, the surrounding vital structures such as the ICAs and optic canals, and the extensive infiltrating tumor.

### 3.4. Fluorescence Identification of Vital Structures by the ICG Endoscope

ICG was prepared by the anesthesiologist and administered on demand of the surgeon. In all patients, good identification with respect to the ICAs, cavernous sinus (CS), intercavernous sinus (ICS), and normal pituitary gland was obtained. Intravenous ICG (12.5 mg) was administered via bolus injection and followed by flushing with 10 mL of saline. At 10 to 15 seconds (s) after flushing, the bilateral ICAs were visualized as a strong fluorescent signal through the thin bone of the carotid prominence (Figures 1(c) and 1(e)). A few seconds later, the CS was also visualized. At 30 to 40 s after flushing, the fluorescent signal of ICG from the normal pituitary gland that had been extracapsularly dissected from the tumor came into view (Figures 1(d) and 1(f)). We also used an electromagnetic navigation system to localize the anatomical structures, but ICG endoscopic visualization was far superior to the navigation system, particularly just before opening the sellar floor and in the case of a little bleeding from tumors, because the ICG endoscope allowed for real-time monitoring of the vital structures and displayed the anatomical relationships between the tumor and surrounding structures.

### 3.5. Illustrative Cases

**Figure 2:**
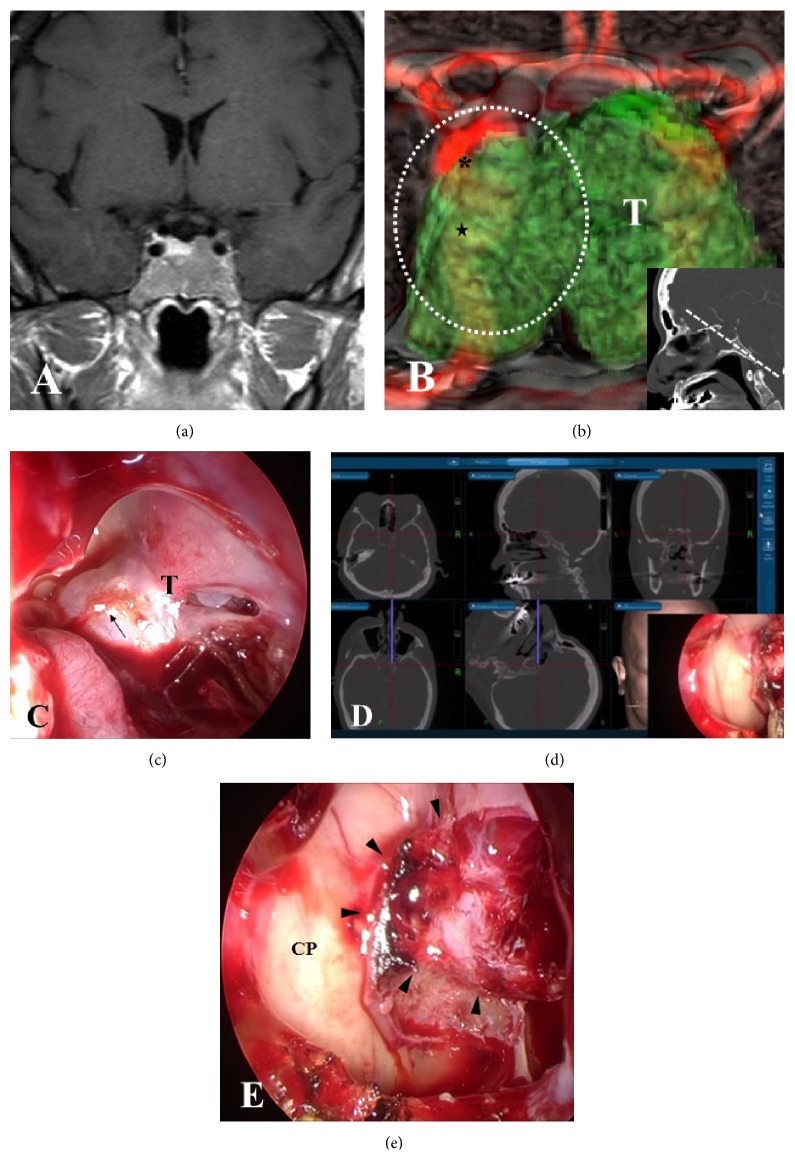
Preoperative image of a fusion model of 3D-CT and MRI and intraoperative endoscopic view in Case 1. (a) Preoperative coronal Gd-enhanced MRI in Case 1 showing a pituitary tumor with extensive invasion into the sphenoidal sinus. (b) Preoperative 3D-CT and MRI fusion model demonstrating that bilateral ICAs (C3 and C4 portions) are buried in the invasive tumor at the anterior plane of the sella (inset) (stars: C4; asterisks: C3). (c) Endoscopic view before tumor resection showing the massive tumor that widely protruded into the sphenoidal sinus. (d) A neuronavigation system demonstrating the locations of the ICAs and the destroyed sella in the endoscopic view (c). CT images on the navigation display show the position of the chip and trajectory of the flexible probe that was placed on the marked position (black arrow) on the endoscopic view (c). (e) Endoscopic view after resection of the tumor showing the widely eroded sellar floor occupied with residual intrasellar tumor and intact bony structures over the ICAs (arrowheads: eroded sellar floor; CP: ICA prominence). T: tumor; the area of white-dashed line: endoscopic view in (c).

*Case 1*. A 49-year-old woman was diagnosed with a growth hormone producing pituitary adenoma 3 years before and she had been treated with an intramuscular injection of octreotide. However, she suffered from persistent nasal congestion and sleep apnea syndrome. Gadolinium- (Gd-) enhanced MRI showed a macroadenoma with inferior extension into the sphenoidal sinus. The sellar floor had been destroyed by the invasive tumor and the bilateral ICAs from the C3 to the C5 portions were completely encased by the tumor (Figure 2(a)). We created the fusion model to make a preoperative plan for how to resect the tumor effectively and safely because it was difficult to identify the exact locations of the ICAs that were buried in the tumor with the 3D-CT model alone. Successive elimination of bony structures on the fusion image of 3D-CT and MRI clearly revealed the location of the bilateral ICAs surrounded by infiltrating tumor (Figure 2(b)). The intraoperative endoscopic view correlated with the images on the fusion model, but the exact location of the ICAs could not be identified due to the widely protruding tumor mass (Figure 2(c)). Thus, the electromagnetic navigation system was used to identify the sellar structures, including the ICAs (Figure 2(d)). Finally, we were able to resect most of the tumor that had extended through the destroyed sellar floor (Figure 2(e)).

**Figure 3:**
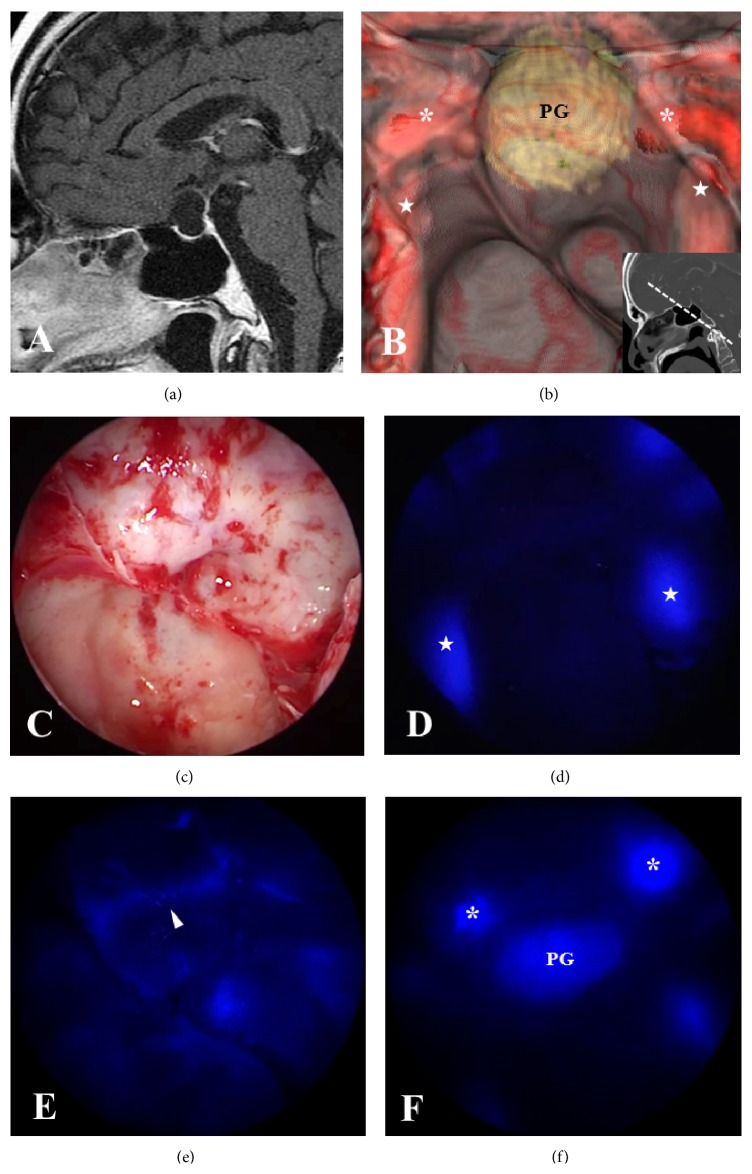
Preoperative image of a fusion model of 3D-CT and MRI and intraoperative endoscopic view along with ICG endoscopic views at each elapsed time in Case 2. (a) Preoperative sagittal Gd-enhanced MRI in Case 2 showing a cystic lesion in the sellar and suprasellar region. (b) A fusion model of 3D-CT and MRI demonstrating an anatomical relationship between bilateral ICAs and the pituitary gland that is flattened at the sellar floor. The bony structures are partially eliminated (asterisks: C3; stars: C4) and the normal pituitary gland (PG). (c) Endoscopic view demonstrating the structures around the sella that correspond to the fusion model. (d, e, f) ICG endoscopic views visualizing bilateral ICAs in the carotid prominences (stars) at the earliest phase, followed by ICS (arrowheads), and the normal pituitary gland (PG) at 5 to 10 s later.

*Case 2*. A 64-year-old man complained of deterioration of visual function and general fatigue. Laboratory studies showed a low plasma level of all adenohypophyseal hormones. Gd-enhanced MRI showed a cystic lesion in the sella (Figure 3(a)). A reconstructed 3D-CT and MRI fusion model allowed localization of the ICAs, optic nerves, and the normal pituitary gland, which were observed through the thinning bone structures (Figure 3(b)). Under the guidance of the 3D-CT and MRI fusion model, we made a preoperative plan and decided how best to open the sellar floor, particularly to expose the upper part of the dura mater because the normal pituitary located in the sellar floor and the anterior-superior part of the sella was determined to be the appropriate site for opening the cyst wall. Intraoperative endoscopic views correlated with the fusion model images (Figure 3(c)). Introduction of the ICG endoscope allowed visualization of the bilateral ICAs 10 s after ICG flushing; the ICS could be seen 20 s later, and the normal pituitary gland could be seen 25 s later (Figures 3(d), 3(e), and 3(f)). The cyst contents were removed, and a part of the cyst wall was removed and used for histological examination. The histological diagnosis was Rathke's cleft cyst. Postoperatively, the patient's visual function improved, although he still required hormone replacement therapy, including hydrocortisone and levothyroxine. Nonetheless, his condition has not deteriorated, nor has diabetes insipidus appeared.

**Figure 4:**
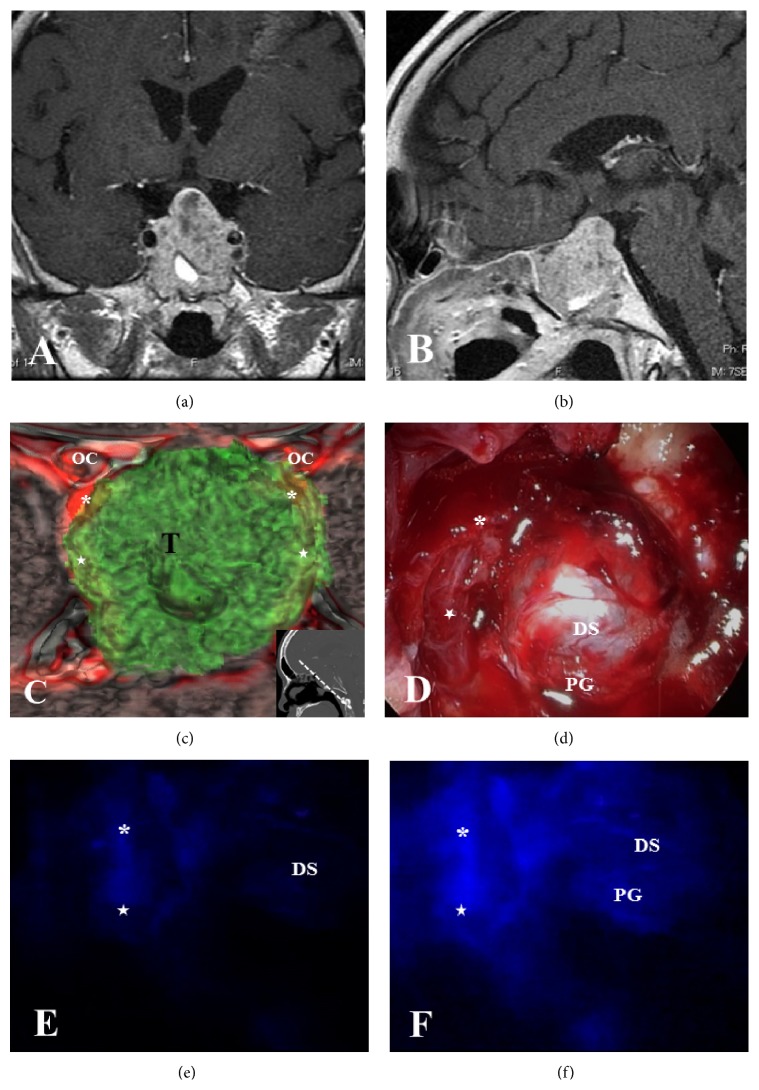
Preoperative image of a fusion model of 3D-CT and MRI and intraoperative endoscopic view and ICG fluorescence views in Case 3. (a, b) Preoperative coronal and sagittal Gd-enhanced MRI in Case 3 showing a pituitary tumor extending into the sphenoidal sinus with involvement of both ICAs by the tumor. (c) Preoperative 3D-CT and MRI fusion model revealing the relationship between the tumor and the vital structures such as the ICAs and optic nerves and the location of the ICAs (asterisks: C3; stars: C4) by eliminating bony structures. (d) Endoscopic view after decompression of the tumor showing residual tumor bilaterally with descending diaphragma sellae (DS) centrally. (e, f) ICG endoscopic views visualizing the right ICA (asterisk: C3; star: C4) under the tumor and the normal pituitary gland (PG) at the posterior part of the diaphragma sellae. T: tumor.

*Case 3*. A 59-year-old man visited our department after demonstrating gradually worsening visual field deficits. Coronal and sagittal Gd-enhanced MRI showed a macroadenoma involving bilateral ICAs with invasion into the sphenoidal sinus and destruction of the sellar floor (Figures 4(a) and 4(b)). Reconstructed 3D-CT and MRI fusion model images revealed the location of the optic canals and bilateral ICAs (Figure 4(c)). However, during the actual ETSS procedure, the extensive tumor made it difficult to identify the exact location of the ICAs that were buried in the tumor. The endoscopic view after decompression of the tumor within the safety zone demonstrated residual tumor bilaterally, with the descending diaphragma sellae situated centrally in the surgical field (Figure 4(d)). To confirm the exact location of the right ICA and the normal pituitary gland, we utilized ICG endoscopic visualization. The right ICA was visualized after ICG administration under the residual tumor (Figure 4(e)), and the fluorescent signal persisted until visualization of the normal pituitary gland, which was observed at the posterior part of the diaphragma sellae (Figure 4(f)). As a result, the tumor was removed as completely as possible without any complications, and the postoperative course was uneventful.

## 4. Discussion

In the current study, we successfully performed ETSS for 35 patients with pituitary tumors and intrasphenoidal sinus tumors using multimodal assistant systems, consisting of a fusion model of 3D-CT and MRI and ICG fluorescence endoscopy. These assistant systems enabled simulating the surgical procedure as a competent guide and visualizing the vital structures such as ICA involved in tumors in real time, resulting in maximum tumor resection without serious complications.

ETSS is highly effective in the resection of pituitary tumors with suprasellar and/or infrasellar extension including midline skull base lesions [1–6]. Introduction of an endoscope in transsphenoidal surgery provides surgeons with a wide panoramic view and the use of multiangled endoscopes provides multidirectional views, facilitating much clearer visualization of the anatomic relationships between the sella and surrounding vital structures, such as the ICAs and the optic nerves. However, their anatomic landmarks are sometimes hidden by bony structures and extensive tumors, which can result in spatial disorientation during ETSS. To avoid any injury to the ICAs, to date, we have adopted a 3D-CT model to help clarify the anatomy of the nasal cavity and paranasal sinuses. With this model, we obtained information on the locations of the ICAs and optic nerves and the anatomic relationship between these structures and the sellar floor. However, in case of tumors infiltrated into paranasal sinus extensively, normal bony structures were destroyed and ICAs were involved in the tumor. In addition, the dura mater and the normal pituitary gland may also be indistinguishable from the invasive tumor. In those cases, we also applied a magnetic navigation system to monitor the exact location of the ICAs in both the cavernous sinus and intracranial subarachnoid space. While a neuronavigation system can direct the field of view to the exact site of interest, the usefulness of the system is limited because some vessels cannot be detected on preoperative MRI scans [13]. Thus, to perform ETSS safely, surgeons must be highly skilled, and multidisciplinary assistant systems for ETSS are required.

The 3D-CT and MRI fusion model in this study facilitates identification of the exact location of important structures, including the ICAs, optic nerves, and Onodi cells, when the surgeon is preparing to perform ETSS. If preoperative planning is performed conventionally with 2D images or a 3D-CT model alone, a considerable amount of effort is required to obtain imaginary images that are as clear as those acquired via 3D reconstruction, in particular, when the tumors have grown extensively in the paranasal sinuses. The 3D-CT and MRI fusion model provides the surgeon with a clearer method of orientation, which is critical for performing ETSS. Furthermore, the model can be optimized for preoperative surgical planning according to the individual anatomy of the patient.

However, there are some cases in which this 3D-CT and MRI fusion model may be less effective. For example, when the sphenoidal sinus is occupied by a downward extending tumor, bony structures, including the carotid prominence, cannot be identified. In such cases, it is very important to accurately identify the ICAs in the early stage of the surgery in order to resect the tumor without injury to the ICAs. To handle this situation, we have applied other methods in combination with this model, such as fluorescence detection of the ICAs by ICG endoscopy. In recent years, endoscope-integrated ICG video angiography has been introduced to assist with confirmation of the patency of vessels hidden from microscopic and plain endoscopic views [12, 13, 15]. ICG is an ideal agent for imaging vessels as it is tightly bound to plasma albumin, has a short half-life, and maintains an acceptable safety profile [14, 18]. In previous reports, the ICAs and the patent CS were clearly detected with ICG endoscopy in real-time and at high resolution. Researchers reported that the ICAs appeared at 10 to 15 s after flushing with ICG; the ICS and CS were seen at 3 to 5 s after ICA detection, and the normal pituitary was seen at 30 s after ICA detection [13–15]. In addition, Litvack et al. reported that adenomas were less ICG-fluorescent than the normal pituitary gland [14]. In the current study, we demonstrated that intraoperative use of ICG endoscopy provides visualization of the exact location of the ICA, CS, and ICS in ETSS. Additionally, we demonstrated that ICG endoscopy enables differentiation between normal pituitary gland tissue and tumor. On the other hand, it is limited in that ICG fluorescent signals persist in solid tissues, so background signals might be high [13]. Therefore, it is important to know what structures are to be inspected and how to interpret the findings. By combining ICG endoscopy with our reconstructed 3D-CT and MRI fusion models, we can operate on sellar and midline skull-based tumors safely by ETSS.

## 5. Conclusions

Preoperative 3D-CT and MRI fusion models along with ICG endoscopy allow for distinct visualization of vital structures, such as the ICAs, in the case of tumors that have extensively infiltrated the sphenoidal sinus. The fusion images are useful for preoperative planning and for clear guidance during ETSS. In addition, the ICG endoscope is a very useful tool for real-time monitoring during ETSS. These multimodal assistant systems may improve the efficiency of ETSS and facilitate maximum tumor resection without complications.

## Figures and Tables

**Table 1:** Characteristics of patients enrolled in the study.

Parameter	Value
Number of patients	35
Female gender (%)	17 (48.6)
Age (years), median (range)	55.5 (16–84)
Type of pathology (% of pituitary lesions)	
Pituitary adenoma	27 (77.0)
Nonfunctioning adenoma	22 (62.9)
PRL producing adenoma	1 (2.9)
GH producing adenoma	4 (11.2)
Craniopharyngioma	3 (8.6)
Rathke's cleft cyst	3 (8.6)
Chordoma	1 (2.9)
Giant cell tumor	1 (2.9)
3D-CT and MRI fusion model construction (%)	35 (100)
ICG visualization in operation (%)	10 (28.6)

3D-CT: three-dimensional computed tomography; MRI: magnetic resonance imaging; ICG: indocyanine green; PRL: prolactin; GH: growth hormone.

The “Value” represents the number of patients.

**Table 2:** Outcome of endoscopic transsphenoidal surgery with multimodal assistant systems in the visual and endocrinological functions. When deterioration of either visual acuity or visual field was recognized, the patient was defined to have worsened visual disturbance. When replacement of more than one pituitary hormone was required compared to preoperative hormonal status, the patient was defined as being a worsened status in the pituitary function.

Preoperative status		Postoperative status		
Pituitary adenoma (*n* = 27)		Improved	No change	Worsened

Visual disturbance (*n*)	14	9	4	1
Hormone deficiency (*n*)	2	0	2	0

Rathke's cleft cyst (*n* = 3)		Improved	No change	Worsened

Visual disturbance (*n*)	1	1	0	0
Hormone deficiency (*n*)	2	0	2	0

Craniopharyngioma (*n* = 3)		Improved	No change	Worsened

Visual disturbance (*n*)	1	1	0	0
Hormone deficiency (*n*)	3	0	2	1
